# Gaze Following and Pupil Dilation as Early Diagnostic Markers of Autism in Toddlers

**DOI:** 10.3390/children8020113

**Published:** 2021-02-05

**Authors:** Raquel Camero, Verónica Martínez, Carlos Gallego

**Affiliations:** 1 Department of Psychology, University of Oviedo, 33003 Oviedo, Spain; uo217941@uniovi.es; 2 Department of Experimental Psychology, Cognitive Processes and Speech Therapy, Complutense University of Madrid, 28223 Madrid, Spain; carlosgallego@ucm.es

**Keywords:** language acquisition, autism, eye-tracker, pseudowords, pupillometry, gaze fixation

## Abstract

*Background*: Children with autism spectrum disorder (ASD) show certain characteristics in visual attention. These may generate differences with non-autistic children in the integration of relevant social information to set the basis of communication. Reliable and objective measurement of these characteristics in a language learning context could contribute to a more accurate early diagnosis of ASD. Gaze following and pupil dilation are being studied as possible reliable measures of visual attention for the early detection of ASD. The eye-tracking methodology allows objective measurement of these biomarkers. The aim of this study is to determine whether measurements of gaze following and pupillary dilation in a linguistic interaction task are potential objective biomarkers for the early diagnosis of ASD. *Method*: A group of 20 children between 17 and 24 months of age, made up of 10 neurotypical children (NT) and 10 children with an increased likelihood of developing ASD were paired together according to chronological age. A human face on a monitor pronounced pseudowords associated with pseudo-objects. Gaze following and pupil dilation were registered during the task These measurements were captured using eye-tracking methodology. *Results*: Significant statistical differences were found in the time of gaze fixation on the human face and on the object, as well as in the number of gazes. Children with an increased possibility of developing ASD showed a slightly higher pupil dilation than NT children. However, this difference was not statistically significant. Nevertheless, their pupil dilation was uniform throughout the different periods of the task while NT participants showed greater dilation on hearing the pseudoword. *Conclusions*: The fixing and the duration of gaze, objectively measured by a Tobii eye-tracking system, could be considered as potential biomarkers for early detection of ASD. Additionally, pupil dilation measurement could reflect differential activation patterns during word processing in possible ASD toddlers and NT toddlers.

## 1. Introduction

From an early age, babies show a preference for maintaining visual contact with their parents and directing their attention towards the human voice and relevant social stimuli [1]. Moreover, it is stated that, at first, typically developing infants pay more attention to the eyes of their interlocutors [2]. Then, from 6–9 months of age, they go on to spend more time paying attention to the mouth, as they begin to specialize in language and build phonological system. After that, between 12 and 15 months, they direct more attention to the eyes [3]. This allows them to learn and develop the keys to social learning. They also fix their attention on the mouth when they do not know the word, or when they hear a foreign word [2], presumably to get support in visual information [4]. The fact that they pay attention to the eyes and the mouth of the people who interact with them is key to learning phonological and lexical references. It allows babies to construct social knowledge, fundamental to their neurological development and the learning of language [5]. Evidence exists that contact and gaze following act as a precursor to the acquisition of overall attention abilities, the imitation and the acquisition of new knowledge and cognitive abilities which are fundamental in language development [6].

Children with ASD show differences with neurotypical children (NT) when attending to socially relevant areas of the face. As a consequence, they do not analyse gestures and social information from others, or social situations. They appear to perform differently when acquiring basic social knowledge that neurotypical children learn easily [1]. This specific impairment for paying spontaneous attention to that which is socially relevant and to the activities of others is present in these children from the first year of life. Children with ASD show a different pattern of social attention which influences their acquisition of language [7]. In fact, one of the first indications of possible ASD is delayed speech development. Therefore, visual attention could serve as a phenotypical characteristic for the identification and diagnosis of children with high ASD likelihood, before reaching one year of age [8].

Recent research has suggested that eye movements and the reactions to verbal/visual stimuli used in eye-tracking methodology could be used as signs or biomarkers of early diagnosis of ASD [9,10,11]. Eye-tracking is a non-invasive and relatively economical methodology that might potentially be used to detect early biomarkers of autism in children of very early ages (even less than 12 months) [12]. However, only when these markers are reliably established and, consequently, early intervention is initiated, will this be translated into a better quality of life for the parents and ASD children. The use of the eye-tracking methodology for the diagnosis of ASD is widely documented [8] even though this methodology is not consistently used to diagnose ASD in the clinical context.

The eye-tracking methodology allows measurement and objectivization of which zones a person directs their attention to during a certain task (gaze following). Studies carried out with this methodology have found that children and adolescents with ASD, in comparison with those who are neurotypical, spend a lot less time paying attention to those areas relevant to social communication, such as the eyes and the mouth [13,14,15]. Furthermore, it has been observed that eye movements in children with a high likelihood of autism, between six and nine months, show significantly lower gaze fixation in comparison with the neurotypical group. Babies that carry out shorter gaze fixations were afterward diagnosed with autism at 36 months of age [16].

Also, it has been observed that two-year-olds diagnosed with ASD show a greater preference for fixing their attention on geometric figures than on human faces [5]. Equally, significant differences have been found in children with ASD, with respect to neurotypical children, in changes in gaze during a word processing task, given that the former do not move their gaze towards an object when they hear the word [17].

Apart from a decrease in gaze following, there exists a different regulation in the autonomic nervous system (ANS) in children with ASD, which may also be contributing to the differences that they show in social processing. Children with ASD seem to have a higher level of activation of the ANS and show an attentional preference for objects rather than people. According to Porges [18], the ANS plays a central role in communication, a key domain that requires substantial support in the ASD phenotype. From a cognitive point of view, an appropriate level in the situation of arousal facilitates the processing of social information. In this sense, it has been observed that an appropriate autonomic state could be associated with social abilities, but when arousal increases, social behaviour is compromised [19,20]. A reliable measure for studying this ANS regulation would be pupil dilation, given that babies are capable of controlling eye movements from four months of age [21]. Pupillometry has been found to be an adequate measure for testing ANS in paediatric and clinical populations, such as individuals with ASD, because it is less invasive and easy to perform [11].

Anderson et al. [22] studied pupil response to images of faces and non-faces in children with ASD and found that these showed pupil constriction as a response to images of children’s faces. However, neurotypical children showed pupil dilation in response to the same stimuli. Years later, the same authors [23], using the same methodology, included a baseline measure, and observed that the group of children with ASD showed greater pupil dilation at that moment in comparison to the neurotypical group. These results are in line with the theory of the existence of a high level of arousal in children with ASD [11] and it has been speculated that acetylcholine, a neurotransmitter in the ANS, is dysregulated in people with ASD [24]. In this same direction, Martineau et al. [25] observed different behaviour patterns in a group of children with ASD compared to neurotypical children on visualising slides of faces, avatars and objects. While the neurotypical group had a significant decrease in pupil size when they had already been shown the stimuli, in the children with ASD high pupil dilation was observed during the entire experimental situation. This seems to indicate atypical functioning of ANS. Other studies also support this hypothesis [12,16,26,27]; a high level of arousal could be atypical, giving rise to more invariant patterns of gaze and visual movements. Furthermore, this variable seems to be related to frequent sleep disruptions which are suffered by children with ASD [28].

This study aims to test the use of eye-tracking methodology as a measure for early detection of ASD in a communicative interaction task. Currently, as far as we know, measures do not exist which allow objectifying and making an early diagnosis of these neurodevelopmental disorders from a linguistic processing task in Spanish. The objective may be considered of great relevance due to the prevalence estimates of ASD in Spain, which is similar to the international rate of 1% [29]. So far, these measures have not been studied in a linguistic interaction task with toddlers. In a study with adults [30], it was found that when neurotypical subjects hear a new word, their pupil dilates significantly compared to the baseline. Therefore, children with typical development, interested in learning language and with a balanced arousal level, are expected to show greater pupillary dilation after listening to a pseudoword compared to children with ASD.

Therefore, the first aim will be to compare the gaze following of children with ASD and neurotypical children (NT) when they hear a pseudoword emitted by a human face using an eye-tracking methodology. This aim is about confirming that children with NT will fix their gaze on the human face a greater number of times, specifically on the eyes, except on hearing the pseudoword, when visual attention will be fixed on the mouth. On the other hand, patterns of visual attention in children with a high likelihood of autism will be more inconsistent, fixing their visual attention a greater number of times on the object and ignoring or paying very little attention to the human face.

A second objective will be to compare the size of pupil dilatation in both groups when they hear the pseudoword. The hypothesis, supported by other studies previously cited, is that the pupil dilation in children with NT will increase when the pseudoword is presented. This is thought to be due to the fact that attention, and cognitive activity in general, increases when having to process a linguistic element, especially if it is unknown. In contrast, in the case of children at risk of having ASD, the greatest dilation will be seen outside the linguistically relevant, because they tend to show preferential gazing towards non-social information as opposed to social information. It has been argued that pupilar dilations in response to cognitive tasks depend on attentional control and they seem to be independent of those produced by emotional arousal [31,32]. It has been assumed that this also happens also when processing the word [30].

## 2. Materials and Methods

### 2.1. Participants

The sample was made up of 20 Spanish toddlers. Of these, 10 participants were identified with a high likelihood of ASD (1 girl and 9 boys), with an age range of between 17 and 24 months (M = 21 and SD = 2.357) and 10 NT individuals (3 girls and 7 boys), with an age range of between 17 and 24 months (M = 20 and SD = 1.944). All of the participants were attending Preschool in Asturias, Spain.

The children with ASD were referred by the Early Attention Unit service, in the location where the referral was made to this specific Unit for the treatment of autism “ADANSI” (Association of people with autism “Silent Children”). The criteria for selection of the children with a high likelihood of autism was: children aged between 17 and 24 months with diagnostic reports of autism from the Neuropediatric Service, and in accordance with the following criteria: significant language delay, scarce visual contact, lack of response when called by name, without hearing or vision problems, low communicative intention and scarcity or lack of capacity to imitate. Furthermore, a protocol of previous evaluation was applied to the entire sample to confirm inclusion in the ASD group. This consisted of three tests: the Revised M-CHAT questionnaire (M-CHAT-R/F) for the detection of autism in small children with a follow-up interview [33], the Brunet–Lezine Scale (PY.BL.R) of psychomotor development in early infancy [34], and the Autism Diagnostic Observation Schedule-2 (ADOS-2)—Toddler Module and Module 1 (Spanish version) [35]. The M-CHAT was answered by the caregivers at home before the interview, while ADOS-2 was carried out by one of the authors who has wide experience with this scale. The entire sample of ASD children met the established diagnostic criteria.

The neurotypical sample was taken from the first year of Preschool at an Early Education School in the same location. Inclusion criteria for the comparison group were to score at least below 10 in the ADOS-2 schedule and below 2 in the M-CHAT questionnaire in the absence of any neurological, social, intellectual, sensorial or motor disorder as well as having no first-degree relatives with a previous ASD diagnosis. At the beginning of the school year, the centre sent an information letter to all the families of the children in the course for children of 2–3 years of age. All parents or legal guardians gave their consent to participation in the study.

Table 1 shows the scores of the ASD group and the NT group on the three scales that make up the evaluation protocol for the confirmation of the diagnosis. Both the chronological age of the participants and the global development age on the Brunette–Lezine Scale is in months and all the participants a high possibility of ASD show a global age under that for their chronological age. Furthermore, these show a score for the diagnosis of autism of between 7 and 10 (CSS) in ADOS-2, which indicates a high likelihood of ASD, while the NT participants show a score of between 0 and 4 (CSS). Finally, in the M-CHAT questionnaire, the total scores of the ASD group range between 8–20, which indicates a high possibility of ASD. This ranges from 0–1 in the NT group.

The research design was approved by the Ethics Committee for Research of the University of Oviedo. The study was developed in accordance with the code of ethics of the World Medical Association (Declaration of Helsinki) for experiments involving human subjects in research and the Spanish Law for Personal Data Protection (15/1999 and 3/2018) principles.

### 2.2. Procedure

Gaze following (fixing and duration of gaze) and pupil dilation of the participants, measured through the pupil diameter were registered during the task. This emulated a communicative situation of acquisition of language in which the emission of words by a human face in association with objects was observed. In a video projected on a screen, a real face was presented which said a pseudoword at the same time as a drawing of a pseudo-object (non-existent invented object) appeared.

The task consisted of nine trials, where the first two were for training purposes. Each of these consisted of a video that began with a blue screen, a neutral colour that does not influence the child’s pupil dilation, and a fixation point to direct the child’s attention to the centre of the screen. This point, which was maintained for two seconds, corresponds to the baseline of the task. Next, a pseudo-object appeared and emitted an attention-getting sound while remaining in the centre of the screen. When the object remained still, a female face appeared which asked the question: “*What is that*?” with happy and surprised intonation. The face was the only visible part of the body. Immediately following this, the face said the name of the pseudo-object (a pseudoword) with adult-directed natural speech. After hearing the pseudoword, the image of the pseudo-object remained on the screen for two seconds. This was supposed to be the fading and processing time of the pseudoword. After this, the drawing of the pseudo-object disappeared and only the face remained, saying “*It’s gone! And what is it called*?” The face was maintained for another two seconds.

To record information, an eye-tracker apparatus, Tobii Spectrum 600 Hz, was used. The participants sat in the laps of their parents in front of a 16” monitor with a panoramic aspect ratio of 16.9 in a dark soundproof room. Their central vision was lined up with the centre of the monitor, at a distance of 60 cm between the eye and the monitor. Once the participant was in place, a calibration of 5 points was carried out through colourful and attractive cartoons. This way, the luminosity was controlled to ensure that changes in pupil dilatation were due to the task itself and not due to changes in the light. To do this, a photometer MASTECH MS6612 was used, with the criterion that luminosity did not pass 110 lumens.

**Figure 1:**
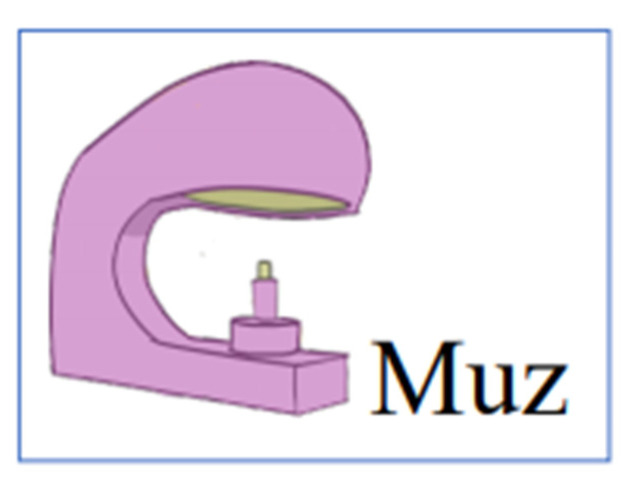
Pseudoword associated with its pseudo-object.

A group of nine pseudowords was selected from a list of test items MEMOFON [36]. Of these, two were for training purposes (*muz and norba*). The pseudowords were differentiated by their complexity both in number and in the type of component syllables. Therefore, two monosyllabic pseudowords were selected, one phonologically simpler with a consonant + vowel + consonant pattern (CVC) (*sel*) and another more complex one with a closed syllable (*tron*). Two pseudowords with two syllables were selected, one more simple (*sina*) and another more complex, since it contains an inverse syllable (*pamul*). Another three pseudowords of three syllables were also selected, two easier (*bésica* and *gapata*) and another more phonologically complex one (*calcemar*). Each pseudoword was presented in association with a drawing of the pseudo-object, an invented object. The pseudo-objects were designed specifically for the experiment and were randomly associated with the pseudowords. In Figure 1, an example of a pseudoword associated with a pseudo-object can be seen.

**Figure 2:**
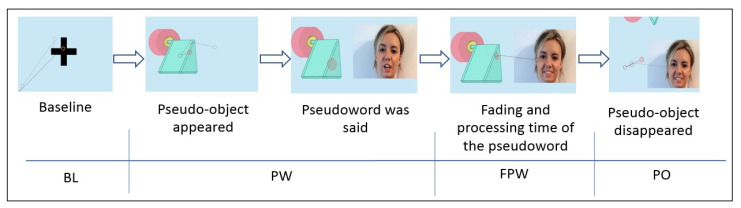
Sequence of a trial.

Once the pseudowords were associated with the pseudo-objects, the order in which the task stimuli were shown to the different participants was random. Figure 2 represents the sequence of one of the trials. The first moment of the sequence corresponded to the baseline (BL) register; the second and third ones corresponded to the moment of the presentation of the pseudoword (PW); the fourth moment was the period of time in the fading of the pseudoword (FPW) and the last sequence of the video was when the pseudo-object first, and later the human face, disappeared from the screen (PO).

The appearance time of the pseudo-objects and the waiting time between the continuation of the image of the object and the production of the pseudoword was determined based on a pilot study of NT toddlers aged between 18 and 30 months. Here, the same pseudo-objects and pseudowords were used. The times obtained were taken as reference criteria since, in the scientific literature, times are not clearly established for a linguistic processing task using eye-tracking methodology at such an early age.

During the entire task, pupil dilation was registered and measured in millimetres every two milliseconds, as well as gaze and the areas of interest on which gaze was fixed: the pseudo-object (AOI 1), the eyes of the face (AOI 2) and/or the area of the mouth pertaining to the face (AOI 3). Data were obtained through the system’s software “Tobii Pro Lab” and included the number and time of gazes at the previously defined areas of interest (AOI), and pupil dilation during the whole task.

### 2.3. Data Analysis

The data obtained were analysed with the programme IBM SPSS Statistics—version 22.0 for Windows. The indices for asymmetry and kurtosis were carried out and a descriptive analysis of the dependent variables (gaze following and pupil measurement), as well as of the variable of classification by chronological age (CA), were carried out. Due to the size of the groups in the study and to the violation of normality and homogeneity variance assumptions, the data were analysed using nonparametric statistics.

In order to confirm whether differences existed between both groups in the gaze following measurement, pairwise Mann–Whitney U tests were used for between-group comparisons. Cliff’s Delta (δ) statistic was chosen as the effect size estimator because it is more appropriate when the homogeneity of variance or normality assumptions are violated. Based on Cohen norms [37], we consider an effect size of 0.2 as a small effect, 0.5 as a medium effect, and 0.8 and upwards as a large effect. These analyses were carried out based on the total number of gaze fixations and the total time of fixations of the participants when looking at the object, the mouth and the eyes. Friedman tests were also carried out to establish within-group differences between AO, and Wilcoxon signed-ranks tests were used for pairwise post hoc comparisons. Bonferroni corrections were applied to adjust the *p*-values for multiple post hoc comparisons.

With regard to the analysis of pupil dilation data, a model used by López-Ornat et al. [30] was followed, thus establishing four periods of measurement. In the first period, during the 400 ms that preceded the start of the trial, the baseline (BL) of pupil dilation of each participant was set, this being a measurement of pupil diameter. At the same time, the point of gaze fixation was determined [38]. After that, pupil dilation was considered during the time of presentation of the pseudoword (PW). This period was of variable duration due to the different lengths of the words, between 650 ms and 1200 ms. The third period corresponded to the following 2000 ms where measurements were registered of the period following the fading of the pseudoword (FPW). The fourth period corresponded to that section of the video when the pseudo-object (PO) and the human face disappeared from the screen, with a duration of two seconds before the next trial began. Only the period of presentation of the pseudoword was of variable duration depending on the length of the pseudoword. Each of these periods included a set of observations taken every two ms. This structure was used for each of the nine trials.

In order to test if there were differences between groups in pupillary diameter throughout the task and in the different periods, Mann–Whitney *U* tests were used. Cliff’s Delta (δ) statistic was used to estimate the effect size. Friedman tests were also carried out to establish within-group differences between periods, and Wilcoxon signed-ranks tests were used for pairwise post hoc comparisons. Bonferroni corrections were applied to adjust the *p*-values for multiple post hoc comparisons.

## 3. Results

No statistically significant differences between the two groups were found with regard to chronological age (*U* = 34.50; *Z* = −1.201; *p* = 0.23; Cliff’s δ = 0.443). As expected, there were statistically significant differences with regard to developmental age (*U* = 6.00; *Z* = −3.412; *p* < 0.001; Cliff’s δ = −0.88).

The skewness index (A) in the number of object fixations (NOF), mouth fixations (NMF), eye fixations (NEF) and in the eye fixation time (EFT) indicated that the distribution data were asymmetrical in the NT group (A = 1.617; 1.138; −2.207; and −3.180, respectively) as well as NMF (A = 1.548) in the ASD group. On the other hand, the kurtosis index (K) indicated non-normal distribution in the NOF (K = 3.515) and the eye fixation time (EFT) (K = 5.362) in the NT group.

Regarding differences between groups, there were statistically significant differences between the ASD group and the NT group in the total number of gaze fixations (*U* = 20.00; Z = −2.27; *p* = 0.023; Cliff’s δ = −0.60) and the total time of fixations (*U* = 0.00; *Z* = −3.78; *p* < 0.001; Cliff’s δ = −0.60).

As shown in Table 2, the performance of the groups in gaze following showed significant differences. The NT participants looked at the eyes a greater number of times (*U* = 0.00; *Z* = −3.79; *p* < 0.001; Cliff’s δ = −1) and for a longer time than the ASD participants (*U* = 0.00; *Z* = −3.78; *p* < 0.001; Cliff’s δ = −1). On the contrary, the ASD group looked at the mouth a greater number of times than the NT group (*U* = 0.00; *Z* = −3.80; *p* < 0.001; Cliff’s δ = 1) and for a longer time (*U* = 0.00; *Z* = −3.78; *p* < 0.001; Cliff’s δ = 1). Similarly, the ASD participants looked at the pseudo-object a greater number of times (*U* = 0.00; *Z* = −3.78; *p* < 0.001; Cliff’s δ = 1) and for a longer time than the NT participants (*U* = 0.00; *Z* = −3.78; *p* < 0.001; Cliff’s δ = 1).

Within-group differences showed statistically significant differences in the number of fixations in the different areas in the NT group (χ^2^ = 20; *p* < 0.001) and also in the ASD group (χ^2^ = 15; *p* = 0.001), but not in the same way. Pairwise post hoc comparisons showed that NT participants made more fixations on the eyes rather than on the object (Z = 2.80; *p* = 0.005; Cliff’s δ = −1), on the eyes rather than on the mouth (Z = 2.81; *p* = 0.005; Cliff’s δ = −1), and on the object rather than the mouth (Z = 2.80; *p* = 0.005; Cliff’s δ = 1). Toddlers with a possible autism diagnosis made more fixations on the object rather than the eyes (Z = 2.80; *p* = 0.005; Cliff’s δ = 1) and on the object rather than the mouth (Z = 2.80; *p* = 0.005; Cliff’s δ = 1). However, there were no significant differences between the number of fixations on the mouth as compared to fixations on the eyes in this group (Z = −0.97; *p* = 0.330; Cliff’s δ = 0.34).

Within-group comparisons also showed statistically significant differences in the time of gaze fixation in the same direction in both groups, NT group (χ^2^ = 20; *p* < 0.001) and ASD group (χ^2^ = 12.20; *p* = 0.002). Pairwise post hoc comparisons showed NT toddlers spent more time looking at the eyes rather than the object (Z = 2.80; *p* = 0.005; Cliff’s δ = −1), at the eyes rather than the mouth (Z = 2.80; *p* = 0.005; Cliff’s δ = −1), and at the object rather than the mouth (Z = 2.80; *p* = 0.005; Cliff’s δ = 1). Toddlers with a possible autism diagnosis spent more time looking at the object rather than the eyes (Z = 2.80; *p* = 0.005; Cliff’s δ = 1) and at the object rather than the mouth (Z = 2.5; *p* = 0.013; Cliff’s δ = 0.610). However, there were no significant differences between the time of gaze fixation on the mouth rather than on the eyes in this group (Z = −0.87; *p* = 0.386; Cliff’s δ = 0.30).

For the calculation of pupil dilation, firstly, a prior pruning of the data was carried out in order to exclude missing data or blinking. Afterward, the mean of pupil dilation was calculated in sections (BL, PW, FPW and PO) for each pseudoword and the total mean of the group of pseudowords was calculated for each section (BL, PW, FPW and PO). Thus, the mean for dilation for the two groups in each section was obtained (Table 3). It can be seen how this was higher in children with possible ASD. In particular, the greater mean for this group was given in section PO, which corresponds to the moment in which the pseudo-object disappears. With respect to the NT group, greater dilation was observed in section PW, the moment when the pseudoword was heard. However, no statistically significant differences between the mean of both groups for pupil dilation (*U* = 32.00; Z = −1.36; *p* = 0.173; δ = 0.20) were observed.

Nevertheless, the within-group comparisons in pupil dilation by periods showed statistically significant differences in the NT group (χ^2^ = 10.80; *p* = 0.013), although there were no differences in the ASD group (χ^2^ = 6.96; *p* = 0.073). Pairwise post hoc comparisons between the four periods in the NT group revealed that pupil dilation was larger during the time of presentation of the pseudoword as compared to that of the pseudo-object and the disappearance of the human face (Z = −2.84; *p* = 0.004; Cliff’s δ = 0.38). Differences in pupil dilation were also close to significance between the time of presentation of the pseudoword as compared with the baseline (Z = −2.30; *p* = 0.021; Cliff’s δ = 0.16), and the time of presentation of the pseudoword as compared with the period following the fading of the pseudoword (Z = −2.32; *p* = 0.020; Cliff’s δ = 0.16), after having been applied Bonferroni correction to adjust the *p*-values for multiple post hoc comparisons (*p* = 0.17). In both cases, pupil dilatation was greater at the time of presentation of the pseudoword.

**Figure 3:**
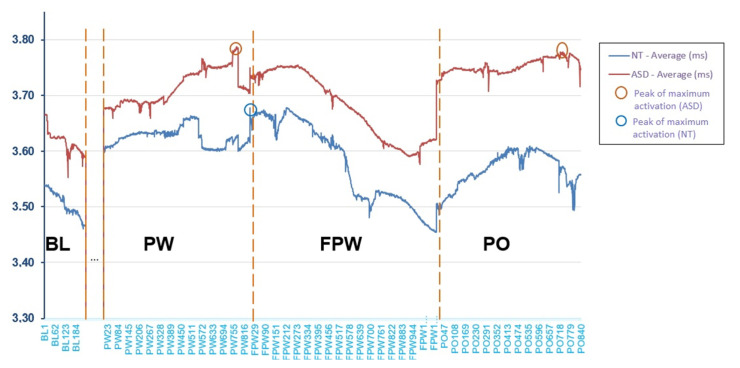
Mean of pupillary dilation throughout the different periods in the entire task (mm).

Figure 3 shows the mean (in millimetres (mm), *y*-axis) of pupil dilation across groups by observation periods during the entire task. The period between BL and PW, marked with dots in Figure 3, has not been analysed because it was not part of the object of study. The *x*-axis shows the trial sequence through observations registered every 20 ms. It can be seen that the ASD participants showed an activation level above that of the NT participants during all periods, although as previously seen, the difference was non-statistically significant. However, the similarity in the shape of the curve indicates that, although there was a slightly higher level of activation, the ASD participants behaved in a similar way to the NT participants. Both groups presented a lower activation level during the baseline (BL) register, at the moment preceding the beginning of the task. Activation increased notably at the beginning of the presentation of the pseudowords (PW) and continued to increase during the task. However, differences may be observed, first, at the end of the time of presentation of the pseudowords, and second, at the maximum peaks of the curve. When the longest pseudowords end, the NT participants showed a drop in their activation that seemed to become stable, while in the ASD group, it continued to increase. On the other hand, the maximum peak of the NT participants was produced when they were processing the pseudoword and the waiting time was going to commence (FPW). In contrast, in the ASD group this was produced almost at the same moment (PW) but also, again, at the moment of the object’s disappearance (PO). Finally, the period of fading of the pseudoword (FPW) lowered the activation of both groups. Then, when the object disappeared and the face said the pseudoword again (PO), activation once again increased in both groups but only in the ASD group did it again reach the peak of maximum activation.

## 4. Discussion

Gaze following and pupil dilation in a linguistic processing task were tested as possible biomarkers for early diagnoses of autism spectrum disorder. Regarding gaze following, the present study has objectively corroborated, using the eye-tracking methodology, that while NT toddlers displayed more numerous and longer fixations on the eye regions than children with a high possibility of developing autism, these displayed more and longer fixations on objects and the mouth regions than NT toddlers. Additionally, ASD toddlers look more and for a longer time at objects rather than eyes and mouth. However, NT toddlers fix their attention on the eyes rather than objects and they pay little attention to the mouth. Thus, while in communicative interaction, NT children spend most of the time looking at the eyes, the children with possible ASD show preference for objects, which could translate into difficulties when integrating social information [5,15]. Therefore, the results obtained are in line with the conclusions already made in other studies [12,15,16,17], suggesting the presence of a non-typical control of attention in ASD, reduced general attention to the eyes and greater attention to non-social elements.

This different use of gaze following as a clue to social reference for learning words could interfere in the acquisition of language, given that eye contact is essential for labelling a referent with a certain word [39]. This different pattern could be attributed to the fusiform gyrus hypoactivation in ASD [13], and it could have later consequences, paying more attention to phonological information than semantic information and social cues [40] and failing to form more robust lexical representations of words [17]. Additionally, it was observed that this atypical pattern in the initiation of joint attention and gaze alternation in ASD, could make the caregiver respond less to a child who does not initiate joint attention [39].

With the results found here, it may be claimed that the measurement of the visual following and attentional preference could be sensitive when differentiating an ASD gaze pattern with a neurotypical gaze pattern. Thus, gaze following measurements related to social attention are good candidates for use as early biomarkers. This may allow us to objectively establish a suspicion or a high likelihood of autism at an early age. This could be quite useful because detection or diagnoses of autism by the Early Attention Unit service are now carried out too late (usually at 30 months) and perhaps it could contribute to distinguishing children with ASD from late talkers or those misdiagnosed with maturational delays [41,42]. With these eye-tracking measurements the diagnoses would no longer depend on subjective clinical judgments, but rather, would provide us with an objective and reliable measure to make solid autism diagnoses.

Regarding the measurement of pupil dilation, the results are not so clear. The ASD group shows a slightly larger average pupil size throughout the task than the NT group. This could suggest that children with ASD show hyper-arousal in the tasks which they must face, which would be in accordance with previous research [26,27,28,43]. A rising level of activation during the task would translate into attention level difficulties that could form the basis of the characteristics that these children show when processing social information in different contexts. Nevertheless, this difference was not large enough to reach a significance level in the complete task. So, no conclusion can be drawn. Other researchers also found no differences in arousal between children with ASD and NT children [44]. This could be attributed to the early age of the participants [27,44,45,46]. Dinalankara et al. [45] observed that the baseline pupil size increased with age, up to four years in NT children, but this pattern was not observed in children with ASD. However, from the age of four, children with ASD had a larger mean baseline pupil size than NT children. These changes with age appear to be due to the increased acceleration of white matter maturation in ASD [45]. Another possible explanation for our results could be the level of possible autism in our participants because it was observed that toddlers with a high risk of ASD presented larger base pupil size in resting than toddlers with a low possibility of ASD [46].

However, an interesting issue arises from within-group pupillary dilation results. There were no significant differences in activation measured through pupillary dilation between periods in the ASD toddlers. Nevertheless, in the NT toddlers, a higher level of activation took place during the hearing (and processing) of the pseudoword, compared to the other periods. This suggests a higher level of active attention in this period. This may confirm the previously formulated hypothesis that pupil dilation in children with NT will increase when the pseudoword is presented, because they are attending to language. While in children with ASD, no larger dilation will be seen in the linguistically relevant, because they would show low selective attention to relevant information for communication. Indeed, in this group higher activation is observed in the final period. It was observed that cognitively relevant pupil dilations are caused by the inhibition of the parasympathetic nervous system and by acetylcholine, which plays an important role in the regulation of attention control [24].

In addition, the NT group’s maximum peak of dilation was found at the end of the processing of the pseudoword, indicating that these children are paying attention and retaining the phonological representation of the pseudoword [30] in working phonological memory, and that they are making a greater cognitive effort at this point. They are ready to learn language and to concentrate their interest on this. In the children with possible ASD, the maximum average value is produced during the disappearance of the pseudo-object (PO), and the maximum peak of dilation occurs at the end of the pseudoword presentation (PW) as in the NT group. However, this also occurs in the period during which the pseudo-object disappears. Two maximum peaks were considered, since the variation between both of them is practically null. These results are not in accordance with what Anderson and Colombo set out in their study [23], since these authors found the maximum peak in the baseline section.

To sum up, in the present study, the eye-tracking methodology was used in an innovative way in a linguistic processing task in children of an early age. It was shown that these types of tests could provide evidence when measuring attention bases in the development of the process of acquisition of language in children, not only after 24 months of age [9] but also before that age. In addition, in comparing NT toddlers with possible ASD toddlers, differences are observed in the development of the pattern of gaze during the acquisition of linguistic abilities which appear to have great diagnostic potential.

These findings should be interpreted from a neuropsychological perspective, since alterations in visual attention are indicative of a state of anomalous neural activation. The results found indicate that indirect, objective measurements of the level of activation, such as number and time of gaze fixations (registered through eye-tracking) are potential candidate biomarkers for diagnostic indicators of the presence of ASD [43]. Even so, it would be necessary to carry out a larger future study of these measurements to refine this technique for non-invasive diagnostic screening. It is easy to administer and economical for the detection of anomalous gaze patterns in children who could have an autism spectrum disorder.

Regarding pupil dilation measurement results, these are not conclusive. Its use as a biomarker diagnostic indicator to identify children with a high likelihood of autism at an early age is not clear. However, it appears to be a hopeful candidate for investigating the differential processing of new words in NT toddlers and possible ASD toddlers. In any case, further research is required and the number and type of stimuli to be processed must be increased.

Finally, despite the encouraging results obtained, some limitations must be mentioned. First, the size of the sample, since these results could not be extrapolated to the entire autism population. Secondly, it is clear that the applied measurements do not allow the establishment of a definitive diagnosis of ASD and, at the moment, the participants are not being longitudinally monitored to ascertain a final diagnostic outcome. Finally, it should be pointed out that the stimuli in the linguistic interaction task were presented in a video and not in a live social situation.

## Figures and Tables

**Table 1:** Scores of participants with autism spectrum disorder (ASD) and neurotypical children (NT) in the three diagnostic tests.

Group	Participant	Brunette–Lezine		ADOS (CSS)	M-CHAT
		Chronological Age	Global Age of Development		
ASD	1	17	10	22 (10)	15
	2	21	18	14 (7)	8
	3	20	12	21 (10)	10
	4	18	9	22 (10)	12
	5	22	18	22 (10)	15
	6	24	18	20 (10)	20
	7	24	16	22 (10)	8
	8	20	18	15 (9)	9
	9	21	12	21 (10)	10
	10	23	12	20 (9)	11
NT	1	18	18	2 (2)	0
	2	20	21	0 (0)	0
	3	20	20	6 (3)	0
	4	17	18	9 (4)	1
	5	22	24	8 (2)	0
	6	24	24	2 (2)	0
	7	20	21	0 (0)	0
	8	20	20	0 (0)	0
	9	19	18	4 (2)	1
	10	20	20	6 (2)	0

**Table 2:** Median (MDN), Interquartile range (IQR), differences between groups, the *p* values, and effect size in number and time of gaze fixation on the areas of interest between ASD and NT.

		ASD		NT				
		MDN	IQR	MDN	IQR	*Z*	*p*	Cliff’s δ
Eyes	NEF	34.50	16.00	210.50	10.00	−3.79	0.000	−1
	EFT	16.00	19.71	157.73	13.28	−3.78	0.000	−1
Mouth	NMF	41.00	45.00	3.00	4.00	−3.80	0.000	1
	MFT	19.17	36.76	1.44	4.17	−3.78	0.000	1
Object	NOF	129.50	45.00	35.00	13.00	−3.78	0.000	1
	OFT	53.54	14.23	26.37	4.51	−3.78	0.000	1

NEF = Number of eye fixations, EFT = Eyes fixation time, NMF = Number of mouth fixations, MFT = Mouth fixation time, NOF = Number of object fixations, OFT = Object fixation time.

**Table 3:** Total mean of global pupil dilation for each of the periods (ms).

Group	Total Mean	BL	PW	FPW	PO
ASD	3.702	3.685	3.711	3.659	3.753
NT	3.583	3.525	3.661	3.578	3.567

BL = Baseline; Number, PW = Time of presentation of the pseudoword, FPW = Period following the fading of the pseudoword, PO = Pseudo-object and the human face disappeared.

## Data Availability

The data presented in this study are available on request from the corresponding author. The data are not publicly available due to ethical principles.

## References

[1] Karmiloff K., Karmiloff-Smith A. (2001). Pathways to Language. From Fetus to Adolescent.

[2] Lewkowicz D.J., Hansen-Tift A.M. (2012). Infants deploy selective attention to the mouth of a talking face when learning speech. Proc. Natl. Acad. Sci. USA.

[3] Tenenbaum E., Sobel D., Sheinkopf S., Malle B., Morgan J. (2015). Attention to the mouth and gaze following in infancy predict language development. J. Child Lang..

[4] Young G.S., Merin N., Rogers S.J., Ozonoff S. (2009). Gaze behavior and affect at 6 months: Predicting clinical outcomes and language development in typically developing infants and infants at risk for autism. Dev. Sci..

[5] Shic F., Macari S., Chawarsk K. (2014). Speech disturbs face scanning in 6-month-olds who develop autism spectrum disorder. Biol. Psychiatry.

[6] D’Souza D., D’Souza H., Johnson M.H., Karmiloff-Smith A. (2015). Concurrent relations between face scanning and language: A cross syndrome infant study. PLoS ONE.

[7] Howard P.L., Zhang L., Benson V. (2019). What can eye movements tell us about subtle cognitive processing differences in autism?. Vision.

[8] Chawarska K., Macari S., Shic F. (2013). Decreased spontaneous attention to social scenes in 6-month-old infants later diagnosed with autism spectrum disorders. Biol. Psychiatry.

[9] Murias M., Mayor S., Davlantis K., Franz L., Harris A., Rardin B., Sabatos-DeVito M., Dawson G. (2018). Validation of eye—Tracking measures of social attention as a potential biomarker for autism clinical trials. Autism Res..

[10] Frazier T.W., Klingemier E.W., Parikh S., Speer L., Strauss M.S., Eng C., Hardan A.Y., Youngstrom E.A. (2018). Development and validation of objective and quantitative eye tracking-based measures of autism risk and symptom levels. J. Am. Acad. Child Adolesc. Psychiatry.

[11] De Vries L., Fouquaet I., Boets B., Naulaers G., Steyaert J. (2020). Autism spectrum disorder and pupillometry: A systematic review and meta-analysis. Neurosci. Biobehav. Rev..

[12] Nyström P., Gliga T., Jobs E.N., Gredebäck G., Charman T., Johnson M.H., Bölte S., Falck-Ytter T. (2018). Enhanced pupillary light reflex in infancy is associated with autism diagnosis in toddlerhood. Nat. Commun..

[13] Dalton K.M., Nacewicz B.M., Johnstone T., Schaefer H.S., Gernsbacher M.A., Goldsmith H.H., Alexander A.L., Davidson R.J. (2005). Gaze fixation and the neural circuitry of face processing in autism. Nat. Neurosci..

[14] Klin A., Jones W., Schultz R.T., Volkmar F.R., Cohen D.J. (2002). Visual fixation patterns during viewing of naturalistic social situations as predictors of social competence in individuals with autism. Arch. Gen. Psychiatry.

[15] de Wit T.C.J., Falck-Ytter T., von Hofsten C. (2008). Young children with Autism Spectrum Disorder look differently at positive versus negative emotional faces. Res. Autism Spectr. Disord..

[16] Wass S.V., Jones J.H., Gliga T., Smith T.J., Charman T., Johnson M.H. (2015). Shorter spontaneous fixation durations in infants with later emerging autism. Sci. Rep..

[17] Chita-Tegmark M., Arunachalam S., Nelson C.A., Tager-Flusberg H. (2015). Eye-tracking measurements of language processing: Developmental differences in children at high risk for ASD. J. Autism Dev. Disord..

[18] Porges S.W., Bauman M.L., Kemper T.L. (2005). The Vagus: A mediator of behavioral and physiological features associated with autism. The Neurobiology of Autism.

[19] Patriquin M.A., Hartwig E.M., Friedman B.H., Porges S.W., Scarpa A. (2019). Autonomic response in autism spectrum disorder: Relationship to social and cognitive functioning. Biol. Psychol..

[20] Benevides T.W., Lane S.J. (2015). A review of cardiac autonomic measures: Considerations for examination of physiological response in children with autism spectrum disorder. J. Autism Dev. Disord..

[21] Verschoor S.A., Spapé M., Biro S., Hommel B. (2013). From outcome prediction to action selection: Developmental change in the role of action-effect bindings. Dev. Sci..

[22] Anderson C.J., Colombo J., Jill S.D. (2006). Visual scanning and pupillary responses in young children with Autism Spectrum Disorder. J. Clin. Exp. Neuropsychol..

[23] Anderson C.J., Colombo J. (2009). Larger tonic pupil size in young children with autism spectrum disorder. Dev. Psychobiol..

[24] Hellmer K., Nyström P. (2017). Infant acetylcholine, dopamine, and melatonin dysregulation: Neonatal biomarkers and causal factors for ASD and ADHD phenotypes. Med. Hypotheses..

[25] Martineau J., Hernandez N., Hiebel L., Roché L., Metzger A., Bonnet-Brilhault F. (2011). Can pupil size and pupil responses during visual scanning contribute to the diagnosis of autism spectrum disorder in children?. J. Psychiatr. Res..

[26] Wagner A.E., Toffanin P., Bas K.D. (2016). The timing and effort of lexical access in natural and degraded speech. Front. Psychol..

[27] Segers M., Bebko J.M., Zapparoli B.L., Stevenson R.A. (2020). A pupillometry study of multisensory social and linguistic processing in autism and typical development. Dev. Psychol..

[28] Moore M., Evans V., Hanvey G., Johnson C. (2017). Assessment of sleep in children with Autism Spectrum Disorder. Children.

[29] Morales-Hidalgo P., Roigé-Castellví J., Hernández-Martínez C., Voltas N., Canals J. (2018). Prevalence and characteristics of autism spectrum disorder among Spanish school-age children. J. Autism Dev. Disord..

[30] López-Ornat S., Karousou A., Gallego C., Martín L., Camero R. (2018). Pupillary measures of the cognitive effort in auditory novel word processing and short-term retention. Front. Psychol..

[31] Gilzenrat M.S., Nieuwenhuis S., Jepma M., Cohen J.D. (2010). Pupil diameter tracks changes in control state predicted by the adaptive gain theory of locus coeruleus function. Cognit. Affect. Behav. Neurosci..

[32] Karatekin C., Couperus J., Marcus D. (2004). Attention allocation in the dual-task paradigm as measured through behavioral and psychophysiological responses. Psychophysiology.

[33] Robins D.L., Fein D., Barton M. (2009). The Modified Checklist for Autism in Toddlers, Revised, with Follow-Up (M-CHAT-R/F).

[34] Josse D., Pereda S. (1997). Brunet Lézine Revisado: Escala de Desarrollo Psicomotor de la Primera Infancia (Brunet Lézine Reviewed: A Psychomothor Development Scale for Infants).

[35] Lord C., Rutter M., DiLavore P.C., Risi S., Gotham K., Bishop S.L. (2012). Autism Diagnostic Observation Schedule, Second Edition (ADOS-2) Manual (Part I): Modules 1–4.

[36] Mariscal S., Gallego C. (2013). La imitación como herramienta para investigar y evaluar el desarrollo lingüístico temprano: Un estudio piloto de repetición de palabras y pseudopalabras (Imitation as a tool for research and assessment of early language development: A pilot study of word and non-word repetition). Rev. Investig. Logop..

[37] Cohen J. (1988). Statistical Power Analysis for the Behavioral Sciences.

[38] Guasch M., Ferré P., Haro J. (2017). Pupil dilation is sensitive to the cognate status of words: Further evidence for non-selectivity in bilingual lexical access. Bilingualism.

[39] Nyström P., Thorup E., Bölte S., Falck-Ytter T. (2019). Joint attention in infancy and the emergence of autism. Biol. Psychiatry.

[40] Norbury C.F., Griths H., Nation K. (2010). Sound before meaning: Word learning in autistic disorders. Neuropsychologia.

[41] Brett D., Warnell F., McConachie H., Parr J.R. (2016). Factors affecting age at ASD diagnosis in UK: No evidence that diagnosis age has decreased between 2004 and 2014. J. Autism Dev. Disord..

[42] Fernald A., Marchman V.A. (2012). Individual differences in lexical processing at 18 months predict vocabulary growth in typically developing and late-talking toddlers. Child Dev..

[43] Del Valle Rubido M., Hollander E., McCracken J.T., Shic F., Noeldeke J., Boak L., Khwaja O., Sadikhov S., Fontoura P., Umbricht D. (2020). Exploring social biomarkers in high-functioning adults with autism and Asperger’s versus healthy controls: A cross-sectional analysis. J. Autism Dev. Disord..

[44] Fan X., Miles J.H., Takahashi N., Yao G. (2009). Abnormal transient pupillary light reflex in individuals with autism spectrum disorders. J. Autism Dev. Disord..

[45] Dinalankara D.M.R., Miles J.H., Takahashi T.N., Yao G. (2017). Atypical pupillary light reflex in 2–6-year-old children with autism spectrum disorders. Autism Res..

[46] Kercher C., Azinfar L., Dinalankara D.M.R., Takahashi T.N., Miles J.H., Yao G. (2020). A longitudinal study of pupillary light reflex in 6- to 24-month children. Sci. Rep..

